# Genomic selection of crossing partners on basis of the expected mean and variance of their derived lines

**DOI:** 10.1371/journal.pone.0188839

**Published:** 2017-12-04

**Authors:** Tanja Osthushenrich, Matthias Frisch, Eva Herzog

**Affiliations:** Institute of Agronomy and Plant Breeding II, Justus Liebig University, Heinrich-Buff-Ring 26-32, 35392 Giessen, Germany; Institute of Genetics and Developmental Biology Chinese Academy of Sciences, CHINA

## Abstract

In a line or a hybrid breeding program superior lines are selected from a breeding pool as parental lines for the next breeding cycle. From a cross of two parental lines, new lines are derived by single-seed descent (SSD) or doubled haploid (DH) technology. However, not all possible crosses between the parental lines can be carried out due to limited resources. Our objectives were to present formulas to characterize a cross by the mean and variance of the genotypic values of the lines derived from the cross, and to apply the formulas to predict means and variances of flowering time traits in recombinant inbred line families of a publicly available data set in maize. We derived formulas which are based on the expected linkage disequilibrium (LD) between two loci and which can be used for arbitrary mating systems. Results were worked out for SSD and DH lines derived from a cross after an arbitrary number of intermating generations. The means and variances were highly correlated with results obtained by the simulation software PopVar. Compared with these simulations, computation time for our closed formulas was about ten times faster. The means and variances for flowering time traits observed in the recombinant inbred line families of the investigated data set showed correlations of around 0.9 for the means and of 0.46 and 0.65 for the standard deviations with the estimated values. We conclude that our results provide a framework that can be exploited to increase the efficiency of hybrid and line breeding programs by extending genomic selection approaches to the selection of crossing partners.

## Introduction

In each cycle of a line or a hybrid breeding program, lines are selected which serve as the parents of the crosses from which the base population of the next breeding cycle is derived. However, not all possible crosses between the superior lines of a cycle can be made and evaluated due to limited resources. The decision which parental lines to cross is therefore an essential factor that determines the selection gain in a breeding program.

The usefulness of a cross [1] is defined as *U* = *μ* + *iσ*_*g*_*h*, where *μ* is the expectation and *σ*_*g*_ the standard deviation of the genetic values of the lines derived from the cross, *i* is the selection intensity [2], and *h* the square root of the heritability. The potential of this concept was recognized early, its practical application, however, was hindered by the difficulty of obtaining good estimates for the standard deviation *σ*_*g*_. The prediction of the segregation variance $\sigma_{g}^{2}$ has therefore been a research subject for many years. A recent review of studies using genetic distances, pedigrees or QTL estimates for this purpose was presented by [3].

Bernardo et al. [4] suggested to estimate the variance $\sigma_{g}^{2}$ from QTL effect estimates assuming unlinked loci. This concept was extended to linked loci by Zhong and Jannink [5] who defined, by omitting the square root of the heritability *h* from the equation of the usefulness, the superior progeny value as *s* = *μ* + *iσ*_*g*_. Their approach was developed for recombinant inbred lines derived by single-seed descent (SSD) and uses additive genetic effects estimated by QTL mapping or genome-wide prediction. Several studies investigated with computer simulations the prediction of the genetic variance within a cross [3, 6–8], but fast and versatile analytical solutions for predicting the variance $\sigma_{g}^{2}$ for arbitrary mating schemes have to our knowledge not yet been developed.

Our objectives were to (1) present an analytical derivation of $\sigma_{g}^{2}$ that is based on the expected linkage disequilibrium (LD) between two loci, and that can be used for arbitrary mating systems, (2) provide formulas for the genetic variance $\sigma_{g}^{2}$ in populations of doubled haploid (DH) and SSD lines derived from a cross after *t* generations of intermating, and (3) illustrate the use of the formulas with published data of the nested association mapping (NAM) population in maize [9].

## Materials and methods

### Derivation of $\sigma_{g}^{2}$ based on LD

To derive the superior progeny value *s* = *μ* + *iσ*_*g*_ of the cross of two homozygous lines, we define the random variable *Z* describing the genotypic values of a population of homozygous lines derived from the cross. The expectation of *Z* is

$$\begin{array}{c} \mu =\text{E}\left(Z\right)=\beta_{0}+\;\sum_{c}\;\sum_{j}\;\text{E}\left(Z_{j}\right), \end{array}$$ (1)

and its variance is

$$\begin{array}{c} \sigma_{g}^{2}=\text{var}\left(Z\right)=\;\sum_{c}\;\sum_{j,k}\;\text{cov}\left(Z_{j},Z_{k}\right) \end{array}$$ (2)

where the summation index *c* sums over chromosomes, *j* sums over the loci on a chromosome, and *j*, *k* sums over all locus pairs on a chromosome. *β*_0_ is the intercept in an additive genetic model and the *Z*_*j*_ are random variables that describe the genetic effect of the allele at locus *j*. *Z*_*j*_ can either be two times the additive effect of the maternal allele, which we denote by *g*_*j*_, or two times the effect of the paternal allele denoted by *h*_*j*_. The event space of *Z*_*j*_ is *ω*_*j*_ ∈ Ω_*j*_ = {*g*_*j*_, *h*_*j*_}. The probability that the random variable *Z*_*j*_ takes the value *g*_*j*_ or *h*_*j*_ is

$$\begin{array}{c} P(Z_{j}=g_{j})=P(Z_{j}=h_{j})=1/2 \end{array}$$ (3)

and, hence, the expectation of *Z*_*j*_ is

$$\begin{array}{c} \begin{array}{ccc} \text{E}(Z_{j}) & = & P(Z_{j}=g_{j})g_{j}+P(Z_{j}=h_{j})h_{j}\\ & = & \frac{1}{2}(g_{j}+h_{j}). \end{array} \end{array}$$ (4)

The effects of the maternal and paternal alleles at locus *k* are *g*_*k*_ and *h*_*k*_. For deriving the covariance between the genotypic values at the two linked loci *j* and *k*

$$\begin{array}{c} \text{cov}(Z_{j},Z_{k})=\text{E}(Z_{j}Z_{k})-\text{E}\left(Z_{j}\right)\text{E}\left(Z_{k}\right), \end{array}$$ (5)

we need the expectation of the random variable *Z*_*j*_*Z*_*k*_ with event space *ω*_*j*,*k*_ ∈ Ω_*j*,*k*_ = {*g*_*j*_*g*_*k*_, *g*_*j*_*h*_*k*_, *h*_*j*_*g*_*k*_, *h*_*j*_*h*_*k*_}. To determine the probability of the four events in Ω_*j*,*k*_, we define the conditional probability

qjk=P(Zk=gki|iZj=gj) (6)

that *Z*_*k*_ takes the value *g*_*k*_ under the condition that *Z*_*j*_ takes the value *g*_*j*_, i.e., the probability that the locus *k* carries the maternal gamete under the condition that locus *j* carries the maternal gamete. Using *q*_*jk*_, we have

P(ZjZk=gjgk)=P(Zj=gj)P(Zk=gki|iZj=gj)=12qjk (7)

and for reasons of symmetry

$$\begin{array}{c} P(Z_{j}Z_{k}=g_{j}h_{k})=\frac{1}{2}\left(1-q_{jk}\right) \end{array}$$ (8)

$$\begin{array}{c} P(Z_{j}Z_{k}=h_{j}g_{k})=\frac{1}{2}\left(1-q_{jk}\right) \end{array}$$ (9)

$$\begin{array}{c} P(Z_{j}Z_{k}=h_{j}h_{k})=\frac{1}{2}q_{jk} \end{array}$$ (10)

which can be used to determine the expectation

$$\begin{array}{c} \begin{array}{ccc} \text{E}(Z_{j}Z_{k}) & = & \sum_{\omega_{j,k}}\;P(Z_{j}Z_{k}=\omega_{j,k})\omega_{j,k}\\ & = & \frac{1}{2}q_{jk}(g_{j}g_{k}+h_{j}h_{k})+\frac{1}{2}\left(1-q_{jk}\right)(g_{j}h_{k}+h_{j}g_{k}). \end{array} \end{array}$$ (11)

If Eqs (4) and (11) are inserted into Eq (5), it results

$$\text{cov}(Z_{j},Z_{k})=\frac{1}{2}q_{jk}(g_{j}g_{k}+h_{j}h_{k})+\frac{1}{2}\left(1-q_{jk}\right)(g_{j}h_{k}+h_{j}g_{k})\;-\;\frac{1}{2}\left(g_{j}+h_{j}\right)\frac{1}{2}\left(g_{k}+h_{k}\right)$$ (12)

and, expanding the brackets,

$$\begin{array}{c} \text{cov}(Z_{j},Z_{k})=(\frac{1}{2}q_{jk}-\frac{1}{4})\;(g_{j}g_{k}+h_{j}h_{k}-g_{j}h_{k}-h_{j}g_{k}). \end{array}$$ (13)

From the definition of the conditional probability *P*(*A*|*B*) = *P*(*A*, *B*)/*P*(*B*) it follows that

$$\begin{array}{c} q_{jk}=P(Z_{k}=g_{k},Z_{j}=g_{j})/P(Z_{j}=g_{j}). \end{array}$$ (14)

Using the definition of the LD coefficient

$$\begin{array}{c} D_{jk}=P\left(Z_{k}=g_{k},Z_{j}=g_{j}\right)-P\left(Z_{j}=g_{j}\right)P\left(Z_{k}=g_{k}\right) \end{array}$$ (15)

and that *P*(*Z*_*j*_ = *g*_*j*_) = *P*(*Z*_*k*_ = *g*_*k*_) = 1/2, we can write *q*_*jk*_ as a function of the expected LD between the two loci *j* and *k* as

$$\begin{array}{c} q_{jk}=\frac{1}{2}+2D_{jk}. \end{array}$$ (16)

### Derivations for SSD and DH lines

Eqs 1–16 can be used to determine the superior progeny value *s* in terms of the expected LD for two linked loci. It can be used for arbitrary mating systems that were used to derive the populations of homozygous lines from the initial cross. Prerequisite is that the expected LD coefficient *D*_*jk*_ and, hence, *q*_*jk*_ for the mating system is known or can be derived.

Two major systems used by plant breeders and geneticists to derive inbred lines from a cross are DH lines and recombinant inbred lines developed by repeated selfing with SSD. DH lines are derived from the F_1_ (F_1_-DH), or after *t* generations of random intermating of the F_1_, which we denote by (F_1_)^*t*^-DH. SSD lines are derived from the F_2_, or after *t* generations of random intermating of the F_1_, which we denote by (F_2_)^*t*^-SSD. We present results for *q*_*jk*_ for the (F_1_)^*t*^-DH and (F_2_)^*t*^-SSD mating systems following the approach of [10].

As an F_1_-DH population consists of gametes generated by an F_1_, the probability that such a gamete carries the alleles of one parental line at the loci *j* and *k* is

$$\begin{array}{c} P\left(Z_{k}=g_{k},Z_{j}=g_{j}\right)=\frac{1-r_{jk}}{2}, \end{array}$$ (17)

where *r*_*jk*_ is the recombination frequency between *j* and *k*. Hence, according to Eq 15, the corresponding LD coefficient is

$$\begin{array}{c} D_{jk}=\frac{1-2r_{jk}}{4}. \end{array}$$ (18)

After *t* generations of random mating, the LD coefficient *D*_*jk*_ of the F_1_ population is reduced by the factor (1 − *r*_*jk*_)^*t*^ [2]. It follows for (F_1_)^t^-DH lines

$$\begin{array}{c} D_{jk}=\frac{1-2r_{jk}}{4}{\left(1-r_{jk}\right)}^{t} \end{array}$$ (19)

and

$$\begin{array}{c} q_{jk}=\frac{1}{2}+\frac{1-2r_{jk}}{2}{\left(1-r_{jk}\right)}^{t}. \end{array}$$ (20)

The LD coefficient in SSD lines derived from a population in Hardy-Weinberg-Equilibrium is [11]:

$$\begin{array}{c} D_{jk}=\frac{D_{jk}^{\prime }}{1+2r_{jk}}, \end{array}$$ (21)

where $D_{jk}^{\prime }$ is the LD coefficient in the initial population. The LD coefficient in an (F_2_)^*t*^ population is the same as in a population of (F_1_)^*t*^-DH lines (Eq 19), therefore, for (F_2_)^*t*^-SSD lines

$$\begin{array}{c} D_{jk}=\frac{1}{1+2r_{jk}}\frac{1-2r_{jk}}{4}{\left(1-r_{jk}\right)}^{t} \end{array}$$ (22)

and

$$\begin{array}{c} q_{jk}=\frac{1}{2}+\frac{1-2r_{jk}}{2-4r_{jk}}{\left(1-r_{jk}\right)}^{t} \end{array}$$ (23)

The recombination frequency *r*_*uv*_ can be derived from an arbitrary mapping function. For example, using Haldane’s mapping function [12], the recombination frequency between the map positions that correspond to *Z*_*j*_ and *Z*_*k*_ is

$$\begin{array}{c} r_{jk}=(1-e^{-2|x_{j}-x_{k}|})/2, \end{array}$$ (24)

where *x*_*j*_ and *x*_*k*_ are the map positions of the two loci in Morgan units.

For the estimation of the expectation and the standard deviation of a cross with the presented formulas, the parameter *β*_0_ and the effects *g*_*j*_ and *h*_*j*_ are predicted with a genome-wide prediction model. Further, a linkage map of the markers used for the estimation of the genetic effects is required for calculation of the recombination frequencies.

### Data set for illustration

To illustrate the application of the formulas and to compare our results to results of simulations with PopVar we used publicly available data that was originally generated for the investigation of flowering time in maize [9, 13–15]. The genotypic and phenotypic data used in the present study were downloaded from www.panzea.org. The data comprised two data sets: an association panel of 282 diverse maize inbred lines and the maize NAM population. The NAM population consists of 25 families of 200 recombinant inbred lines that were derived from crosses of the inbred B73 with 25 lines from the association panel. The 25 parental lines were selected from the association panel to represent its diversity [14, 15]. The field experiments for the association panel and the NAM population were conducted in 2006 and 2007 in eight environments within the US. The phenotypic data were best linear unbiased predictors (BLUPs) for each line from these field experiments. Briefly, field spatial correction was applied within each environment. BLUPs for each line were then predicted using a combined mixed model across environments. A detailed description of the field design and the statistical analysis was presented in [15]. We used the data for the traits days to silking (DTS, female flowering) and days to anthesis (DTA, male flowering). We pre-processed the marker data for the available 1100 SNPs. Only polymorphic markers with a maximum of two alleles, less than 10% missing values and a gene diversity of at least 0.1 were used for estimating the genetic effects. Some individuals from the original 200 progeny per cross and a few lines from the association panel were also discarded as they had more than 10% missing marker data. After cleaning, we performed the final analyses for the present study with 258 diverse lines of the association panel as training set and 4641 recombinant inbred lines from the NAM population as validation set. Each NAM family consisted of 183-200 recombinant inbred lines. For both sets, 325 high-quality SNP markers were available. For calculating the recombination frequencies between marker loci, we used the published linkage map based on the NAM population [14]. The linkage map covered a total genome length of 1400 cM, resulting in an average marker density of one marker every 4.3 cM.

### Prediction of *μ* and $\sigma_{g}^{2}$

For calculating marker effects, the association panel was used as training set. Marker effects were calculated with ridge-regression best linear unbiased prediction (RR-BLUP) [16]. The marker effects *u*_*i*_ were thus estimated by solving the mixed-model equation

$$\begin{array}{c} \left(\begin{array}{cc} 1^{\prime }1 & 1^{\prime }Z\\ Z^{\prime }1 & Z^{\prime }Z+\lambda I \end{array})(\begin{array}{c} {\hat{\beta }}_{0}\\ \hat{u} \end{array})=(\begin{array}{c} 1^{\prime }y\\ Z^{\prime }y \end{array}\right) \end{array}$$ (25)

Employing these genetic effects and the marker genotypes of the parental lines, we estimated *μ* and $\sigma_{g}^{2}$ for the families of the NAM population with Eqs 1 and 2. The estimated means and variances were compared with the observed means and variances for DTS and DTA in the NAM families. Effect estimation and estimation of genetic means and variances were implemented in the C programming language.

### Comparison with simulations

In addition to the estimates obtained with Eqs 1 and 2, we estimated *μ* and $\sigma_{g}^{2}$ for DTS and DTA in the NAM families with the simulation software PopVar [7]. We used the same data sets as input for the simulations as for the calculations with the formulas. As PopVar estimates the marker effects and *μ* and $\sigma_{g}^{2}$ in one analysis step, it was not possible to use exactly the same marker effects in the simulations as were used for the analytical approach. We used the implemented RR-BLUP routine of PopVar without the default cross-validation option it offers in order to obtain marker effects as similar as possible to the ones used in the formulas. For each NAM family, we simulated 200 progeny with 25 replications. Computing time required for simulations with PopVar and for estimating the means and variances with Eqs 1 and 2 was assessed using a Linux system with Intel x5670 processors. The calculations were carried out single-threaded.

## Results

**Fig 1:**
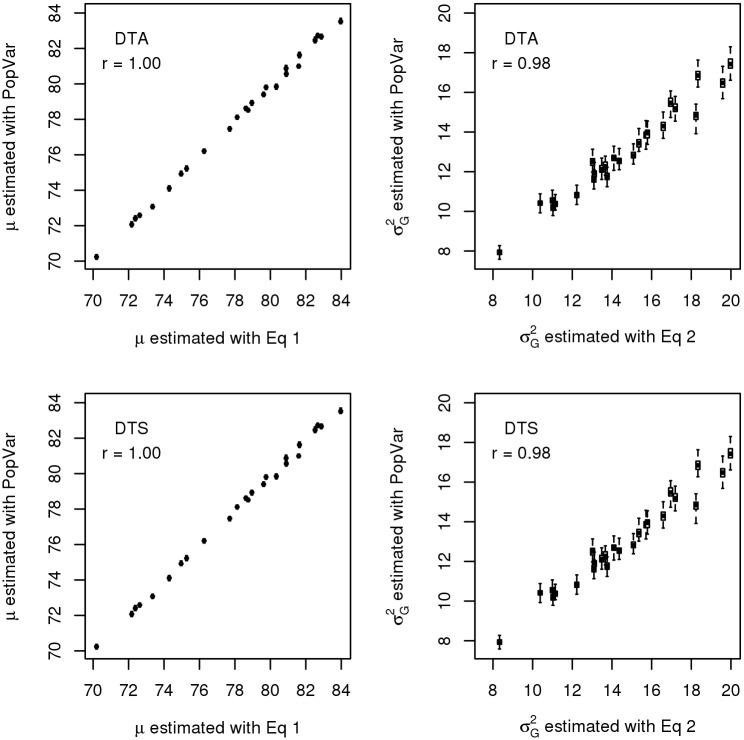
Correlations between the means (left) and variances (right) estimated with Eqs 1 and 2 and the software PopVar. Correlations were calculated for the traits DTA (top) and DTS (bottom) in the maize NAM population.

**Fig 2:**
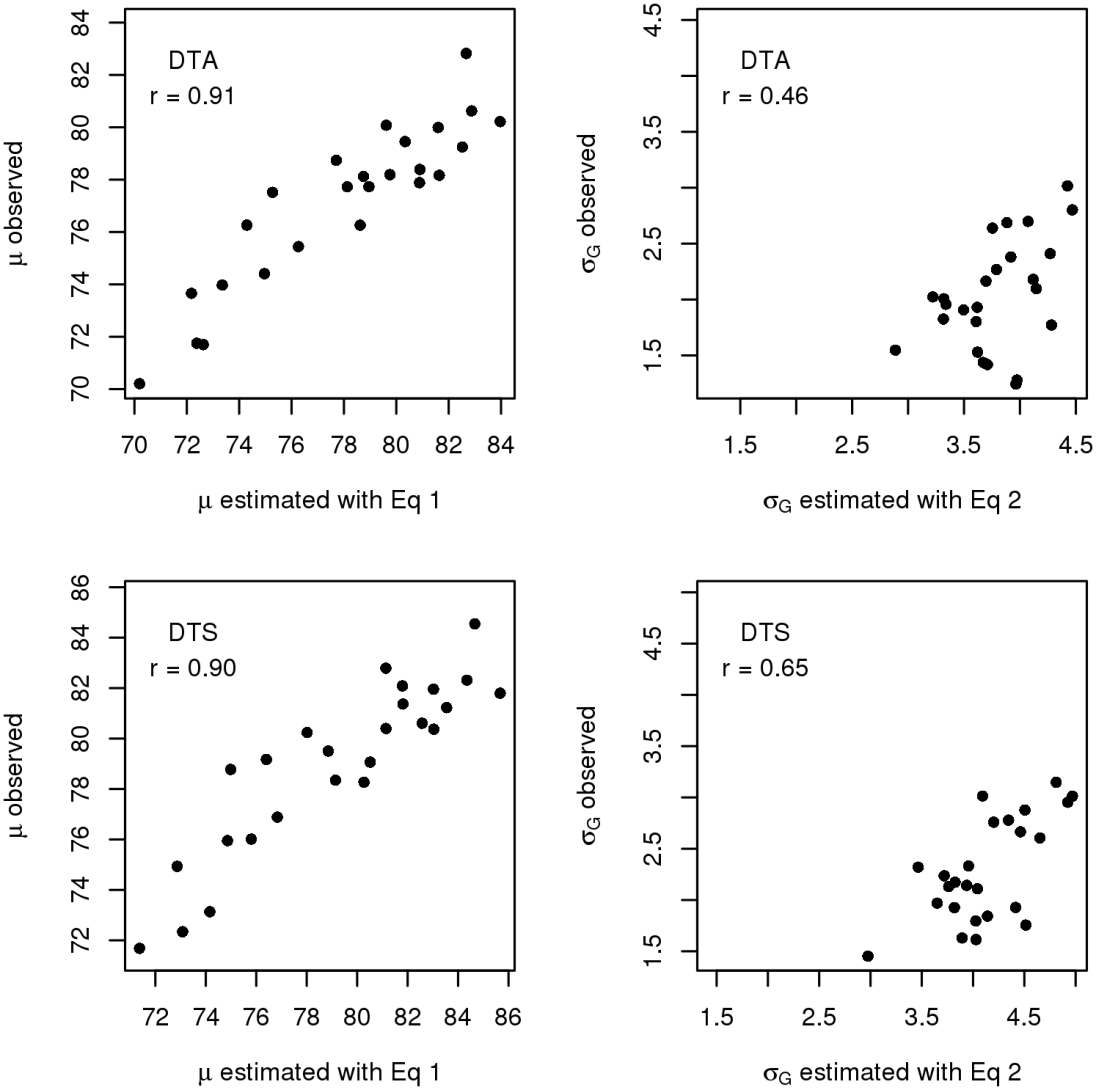
Correlations between the means (left) and standard deviations (right) estimated with Eqs 1 and 2 and the observed parameters from the maize NAM population. The observed parameters were estimated from the observed phenotypic values of the NAM population for the traits DTA (top) and DTS (bottom).

The means and variances estimated with Eqs 1 and 2 showed correlations between 0.98 and 1 with the average of the 25 simulated estimates obtained from PopVar (Fig 1). Estimating *μ* and $\sigma_{g}^{2}$ with Eqs 1 and 2 took 3.3 s and 3.5 s for DTA and DTS, respectively, and obtaining the simulated parameters with PopVar took 46.2 s and 45.5 s, respectively. When the estimates from Eqs 1 and 2 were compared to the observed parameters from the NAM population, the correlations between the observed and estimated means of the crosses for DTA and DTS were 0.90 and 0.91, and the correlations between the observed and estimated standard deviations were 0.46 for DTA and 0.65 for DTS (Fig 2). The estimated standard deviations tended to overestimate the observed standard deviations by factors 1.5-3.

## Discussion

### Differentiation to previous approaches

Zhong and Jannink [5] suggested to assess the value of a cross by its superior progeny value *s* = *μ* + *iσ*_*g*_, where *μ* is the expectation of the genotypic values of the recombinant inbred lines derived by SSD from the cross and $\sigma_{g}^{2}$ is the variance of the genotypic values. *μ* and *σ*_*g*_ were defined in terms of *α*_*i*_, which denotes half of the difference between the two homozygous QTL effects at a locus. This parameterization has the advantage that it can be directly applied for effect estimates from QTL mapping in bi-parental populations. In our notation, the absolute value of the *α*_*i*_ can be expressed as |*g*_*u*_ − *h*_*u*_|/2. Our Eq 2 corresponds to Eq 3 of [5], and is equivalent if employed with an LD coefficient *D*_*uv*_ for F_2_-SSD lines. Our notation has the advantage that the allele effect estimates from genome-wide prediction approaches can be directly used without re-parameterization to *α*_*i*_, and that the formulas also can be applied when effects for multiple alleles were estimated. This is important for applications in which several SNPs are combined to haplotypes.

The analogy between [5] and our approach ends after the authors of [5] presented their Eq 3. For their further derivations, they consider a population of recombinant inbred lines derived from the cross of two inbred lines and they investigate the question which pair of recombinant inbred lines from this population should be crossed to obtain the maximum superior progeny value *s*. The situation we investigate is different. Our approach is not restricted to selection of crossing partners from a set of recombinant inbred lines which were derived from a bi-parental cross. Rather, our approach is targeted at choosing the optimal crossing partners from a set of lines that can be selected from multiple families of a certain cycle of a breeding program to be used as crossing parents for the inbred lines of the subsequent cycle.

As an extension to the approach of [5], our formulas can be flexibly adapted to arbitrary mating systems. Specifically, we presented derivations for DH lines which are used for line development in many breeding programs. Our formulas are also applicable in situations in which several generations of intermating might be required, e.g. if newly introduced diversity needs to be recombined, or if rare transgressive segregants are desired. Moreover, they can be used with arbitrary mapping functions. We therefore believe that our approach has the potential to make the concept of superior progeny value more versatile and applicable in a wider range of crossing scenarios.

In summary, in comparison to the approach of [5], we provide an alternative derivation for the genetic variance of a crossing population that can be used for arbitrary mating systems for which the LD coefficient *D*_*uv*_ can be determined. As an advancement, we provide extensions for DH lines and for an arbitrary number of intermatings before deriving inbred lines by either DH or the SSD. Further, we provide an extension from recombinant inbred lines originating from a single bi-parental cross to sets of inbred lines derived from multiple families from a breeding pool with different parents. We suggest to apply the formulas to select crossing partners from such sets of inbred lines as parents for the subsequent breeding cycle.

### Comparison with PopVar simulations

Bernardo [6] and a series of subsequent investigations [3, 7, 8] suggested to use computer simulations with marker effects estimated by genome-wide prediction models to predict the genetic variance $\sigma_{g}^{2}$ within a cross. Genetic simulations are modeling the recombination along chromosomes, the distribution of the recombined chromatids to gametes, and the union of gametes. The mathematical model using Haldane’s mapping function, which was used in our derivations, is equivalent to the simulation of recombination with a count-location process, using a Poisson distribution for the number of crossovers on a chromosome, and a uniform distribution of the location of crossovers [17].

The software PopVar [3] uses a *χ*^2^ distribution for the number of crossovers on a chromosome and a uniform distribution for the location of the crossovers. This difference in the underlying models is expected to contribute to the differences in the genetic variances predicted with the two approaches in our example data set for traits DTA and DTS (Fig 1). Moreover, due to the implementation of PopVar and the resulting user options, it was not possible to use exactly the same marker effects for estimating the means and variances with PopVar and the derived formulas. However, given that the same mathematically equivalent ridge-regression model was used in both approaches, we expect the differences in marker effects to be negligible. Despite of some small numerical differences in the estimated variances, the two models yielded to a large extent similar results, with correlations of 1 for the mean, and 0.98 for the genetic variances (Fig 1). This could be expected, as both our formulas and PopVar rely on similar assumptions.

However, the major advantage of analytical approaches compared to simulations is speed. In our example data set, only 325 SNP markers were employed, and the analytical approach was about ten times faster as the simulations. It can be expected that the time advantage will be considerably greater with data sets that comprise a greater number of markers.

We conclude that our formulas provide analytical results for the means and variances that are highly correlated to the simulation results, but can be computed more quickly.

### Prediction of genetic variances of the flowering time data set

The accuracy of predicting genetic means and variances obtained when applying our formulas to the flowering time data set is limited by the accuracy of the genetic effect estimates.

In addition to homoscedastic ridge regression, we estimated the marker effects also by using heteroscedastic models [18], but no substantial differences in the results were observed (results not shown).

In data sets from breeding programs, a high marker density is not necessarily required to obtain high accuracies of genomic prediction [19, 20]. This is often attributed to high levels of LD and relatedness in breeding pools. In contrast, here we used a diversity panel consisting of maize lines of different origin as training set. Due to the lower expected LD in such a data set, the number 325 markers seems low for genome-wide prediction.

The number of genotypes in the training set seems also low. In contrast to genetically narrow material from a breeding pool, the number of alleles in the diversity panel is expected to be greater. Hence, the number of replications for each allele in the training set is lower in a diversity set than in material from a breeding program.

Despite of these described properties of investigated data set, the correlations between the estimated and observed means of the crosses for DTA and DTS were 0.90 and 0.91, and the correlations of the estimated standard deviations were 0.46 and 0.65 (Fig 2). We think that these correlations are sufficiently high to create a ranking of crosses based on their superior progeny values *s*. With this ranking, a superior fraction of crosses could be identified and further evaluated to determine the crosses with the highest performance in field trials.

We expect that in breeding pools with longer LD stretches and less allelic variation in combination with a higher marker density, a more precise effect estimation can contribute to greater correlations between estimated and observed segregation variances. We plan further investigations with data sets from breeding programs in sugar beet.

### Application in breeding programs

Due to the comparatively low computation time and versatility with respect to the method of marker effect estimation as well as mating system, the presented formulas can be applied in a wide range of breeding programs. Breeders working in smaller breeding programs for commercially less important crops might consider it a constraint that a linkage map for the markers used for genomic prediction must be available for calculating recombination frequencies. However, the investment might be worthwhile, as a more precise prediction of superior crosses will not only increase selection gain, but also allow for a more efficient allocation of resources.

A possible application scheme for our formulas is outlined as follows. In a breeding program the marker genotypes as well as the performance data for lines selected as crossing parents for the next cycle are routinely available. These data can be used to estimate genetic effects for the markers. Areas of application include prediction of line *per se* performance in line breeding programs, as well as prediction of testcross performance in hybrid breeding programs. On basis of the marker effect estimates from genome-wide prediction models, the means and standard deviations for each cross are predicted.

The relative superiority of the crosses would depend on the ratio of means and standard deviations, as was also pointed out by [5]. In plant breeding programs, it is very common to recombine best-by-best rather than constantly introducing novel variation from genetic resources with poor agronomic properties and adaptation. This implies that the major proportion of the crosses can be expected to have similar cross means with a low variance of the means. Consequently, the genetic variance gains importance as a decision criterion which crosses to make. This holds even more true if we assume that most elite lines are fixed for the same superior alleles, and that a negative covariance might exist between *μ* and $\sigma_{g}^{2}$ [5]. In this case, maintaining genetic variance in the breeding pool is a constant challenge in order to guarantee selection gain in the longer term.

The estimation of the genetic variance could also provide a guideline for the allocation of resources to specific crosses. Consider again the superior progeny value *s* = *μ* + *iσ*_*g*_. If we compare two crosses of elite lines with the same expectation *μ*, the difference in selection gain solely depends on the segregation variance $\sigma_{g}^{2}$ if the same number of progeny is generated and the same selection intensity *i* is applied. However, as the relation between $\sigma_{g}^{2}$ and *i* is a multiplicative one, a cross with a moderately higher genetic variance can result in considerably larger selection gain if the selection intensity is increased. Thus, it makes more sense to invest resources and generate more progeny in crosses with higher segregation variance.

In this context, some consideration should be given to the fact that while the magnitude of correlations between estimated and observed means and variances seems reasonable and useful, the estimated standard deviations tended to systematically overestimate the observed values (Fig 2). This might reduce the efficiency of a breeding program. If the systematic upward bias should in general be so large that it considerably changes the relative magnitude of the segregation variance in comparison to the mean, breeders might invest too many resources in terms of family size without any return on investment. It is possible that the upward bias is in part due the fact that the formulas give us the expected value for an infinite population size, while we compared them to observed values from finite populations which might not realize the full potential of segregation variance. However, this is not likely, as our results were close to the simulation results which were also based on finite population sizes. Further possibilities might be overestimation of the actual recombination frequencies by the mapping function, or the choice of the genome-wide prediction model and the resulting marker effect shrinkage [7].

We still think that it is more efficient to plan the size of the single families within breeding programs based on estimates of the segregation variance than to simply create many small families with the same number of progeny, which is common practice in many plant breeding programs. Open research questions in the field of breeding applications therefore comprise breeding designs that include an optimum family size and selection intensity based on estimated segregation variance. For this goal, the presented approach provides a fast and easy-to-use basis.

## References

[1] Schnell F, Utz H. F_1_-Leistung und Elternwahl in der Züchtung von Selbstbefruchtern. In: Bericht über die Arbeitstagung der Vereinigung österreichischer Pflanzenzüchter. Bundesversuchsanstalt für alpenländische Landwirtschaft Gumpenstein; 1975. p. 243–248.

[2] Falconer DS, Mackay TFC. Introduction to quantitative genetics. 4th ed Uk, Longman: Harlow; 1996.

[3] Mohammadi M, Tiede T, Smith KP. PopVar: A genome-wide procedure for predicting genetic variance and correlated response in biparental breeding populations. Crop Science. 2015;55:2068–2077. doi: 10.2135/cropsci2015.01.0030

[4] Bernardo R, Moreau L, Charcosset A. Number and fitness of selected individuals in marker-assisted and phenotypic recurrent selection. Crop Science. 2006;46:1972–1980. doi: 10.2135/cropsci2006.01-0057

[5] Zhong S, Jannink JL. Using quantitative trait loci results to discriminate among crosses on the basis of their progeny mean and variance. Genetics. 2007;177:567–576. doi: 10.1534/genetics.107.075358 17660556 10.1534/genetics.107.075358 PMC2013701

[6] Bernardo R. Genomewide selection of parental inbreds: Classes of loci and virtual biparental populations. Crop Science. 2014;54:2586–2595. doi: 10.2135/cropsci2014.01.0088

[7] Tiede T, Mohammadi M, Smith K. PopVar: genomic breeding tools: genetic variance prediction and cross-validation. R package version. 2015;1.2.

[8] Lian L, Jacobson A, Zhong S, Bernardo R. Prediction of genetic variance in biparental maize populations: Genomewide marker effects versus mean genetic variance in prior populations. Crop Science. 2015;55:1181–1188. doi: 10.2135/cropsci2014.10.0729

[9] Flint-Garcia SA, Thuillet AC, Yu J, Pressoir G, Romero SM, Mitchell SE, et al Maize association population: A high-resolution platform for quantitative trait locus dissection. The Plant Journal. 2005;44:1054–1064. doi: 10.1111/j.1365-313X.2005.02591.x 16359397 10.1111/j.1365-313X.2005.02591.x

[10] Frisch M, Melchinger AE. Variance of the parental genome contribution to inbred lines derived from biparental crosses. Genetics. 2007;176:477–488. doi: 10.1534/genetics.106.065433 17409089 10.1534/genetics.106.065433 PMC1893034

[11] Cockerham CC, Weir BS. Descent measures for two loci with some applications. Theoretical population biology. 1973;4:300–330. doi: 10.1016/0040-5809(73)90013-0 4747657 10.1016/0040-5809(73)90013-0

[12] Haldane J. The combination of linkage values and the calculation of distances between the loci of linked factors. Journal of Genetics. 1919;8:299–309.

[13] Yu J, Holland JB, Mcmullen MD, Buckler ES. Genetic Design and Statistical Power of Nested Association Mapping in Maize. Genetics. 2008;178:539–551. doi: 10.1534/genetics.107.074245 18202393 10.1534/genetics.107.074245 PMC2206100

[14] McMullen MD, Kresovich S, Sanchez Villeda H, Bradbury P. Genetic Properties of the Maize Nested Association Mapping Population. Science. 2009;325:737–740. doi: 10.1126/science.1174320 19661427 10.1126/science.1174320

[15] Buckler ES, Holland JB, Bradbury Peter. The Genetic Architecture of Maize Flowering Time. Science. 2009;325:714–718. doi: 10.1126/science.1174276 19661422 10.1126/science.1174276

[16] Meuwissen T, Hayes B, Goddard M. Prediction of total genetic value using genome-wide dense marker maps. Genetics. 2001;157:1819–1829. 11290733 10.1093/genetics/157.4.1819 PMC1461589

[17] Maurer HP, Melchinger AE, Frisch M. Population genetic simulation and data analysis with Plabsoft. Euphytica. 2008;161:133–139. doi: 10.1007/s10681-007-9493-4

[18] Hofheinz N, Frisch M Heteroscedastic Ridge Regression Approaches for Genome-Wide Prediction With a Focus on Computational Efficiency and Accurate Effect Estimation. G3: Genes, Genomes, Genetics. 2014;4:539–546. doi: 10.1534/g3.113.010025 24449687 10.1534/g3.113.010025 PMC3962491

[19] Hofheinz N, Borchardt D, Weissleder K, Frisch M. Genome-based prediction of test cross performance in two subsequent breeding cycles. Theoretical and Applied Genetics. 2012;125:1639–1645. doi: 10.1007/s00122-012-1940-5 22814724 10.1007/s00122-012-1940-5

[20] Zenke-Philippi C, Thiemann A, Seifert F, Schrag T, Melchinger A, Scholten S, et al Prediction of hybrid performance in maize with a ridge regression model employed to DNA markers and mRNA transcription profiles. BMC Genomics. 2016;17:262 doi: 10.1186/s12864-016-2580-y 27025377 10.1186/s12864-016-2580-y PMC4812617

